# Supplementary material for: Signs of neuroaxonal injury in preeclampsia—A case control study
Source: PLoS One. 2021 Feb 8;16(2):e0246786. doi: 10.1371/journal.pone.0246786 (PMC7869986; doi:10.1371/journal.pone.0246786)
Supplement: S1 Table — (PDF) [file pone.0246786.s001.pdf]

S1 Table. Concentrations of cerebral biomarkers and association with preeclampsia

	**OR (95% CI)**	**aOR[1] (95% CI)**	**aOR[2] (95% CI)**
**CSF**			
S100B (ug/L)	0.36 (0.26-5.01)	0.25 (0.01-4.46)	0.40 (0.02-10.72)
NSE (ug/L)	0.72 (0.50-1.02)	0.77 (0.55-1.08)	0.66 (0.39-1.12)
tau (pg/ml)	0.99 (0.98.1.00)	0.99 (0.98-1.00)	0.99 (0.98-1.01)
NfL (pg/ml)	1.01 (1.01-1.03)	1.02 (1.00-1.04)	1.01 (1.00-2.02)
**Circulating concentrations**			
S100B (ug/L)*	3.84 (1.07-13.87)	3.62 (0.98-13.38)	1.74 (0.42-7.19)
tau (pg/ml)	1.02 (0.96-1.08)	1.02 (0.95-1.09)	0.98 (0.76-1.25)
NfL (pg/ml)	1.64 (1.09-2.48)	1.70 (1.04-2.78)	1.78 (1.05-3.02)
Albumin ratio	1.30 (0.80-2.11)	1.86 (0.87-3.96)	1.21 (0.77-1.90)

Logistic regression analysis with odds ratios for likelihood of preeclampsia. S100B was measured in serum. The remaining biomarkers were measured in plasma.

[1]Adjusted for parity

[2] Adjusted for body mass index

CSF; cerebrospinal fluid, OR, odds ratio; CI, confidence interval; aOR, adjusted odds ratio; NSE, neuron-specific enolase; NfL, Neurofilament Light Chain

*For an inter-quartile range increase
