# Supplementary material for: Signs of neuroaxonal injury in preeclampsia—A case control study
Source: PLoS One. 2021 Feb 8;16(2):e0246786. doi: 10.1371/journal.pone.0246786 (PMC7869986; doi:10.1371/journal.pone.0246786)
Supplement: S2 Table — (PDF) [file pone.0246786.s002.pdf]

S2 Table. Associations of CSF and circulating concentrations of cerebral biomarkers adjusted for BMI and parity and with a group by biomarker interaction for different associations within the two groups.

**Marker and group**	**OR (95% CI)**	**aOR* (95% CI)**
S100B		
Normal pregnancy	1.17 (0.38-3.65)	1.17 (0.35-3.91)
Preeclampsia	0.79 (0.14-4.40)	0.44 (0.06-3.23)
NSE		
Normal pregnancy	0.34 (0.07-1.44)	0.30 (0.05-1.72)
Preeclampsia	17.89 (1.12-284.85)	56.03 (0.76-4128.71)
tau		
Normal pregnancy	0.22 (0.04-1.14)	0.13 (0.02-0.74)
Preeclampsia	1.46 (0.29-7.23)	1.16 (0.20-6.71)
NfL		
Normal pregnancy	3.57 (1.18-10.78)	3.89 (1.22-12.39)
Preeclampsia	1.86 (0.94-3.71)	2.13 (0.91-5.02)

Values are presented as adjusted odds ratios with 95% confidence intervals.
NSE, Neuron Specific Enolase; NfL, Neurofilament Light Chain; OR, odds ratio, aOR, adjusted odds ratio, CI, confidence interval
*Adjusted for body mass index and parity
