# Supplementary material for: Signs of neuroaxonal injury in preeclampsia—A case control study
Source: PLoS One. 2021 Feb 8;16(2):e0246786. doi: 10.1371/journal.pone.0246786 (PMC7869986; doi:10.1371/journal.pone.0246786)
Supplement: Attachment — Submitted filename: PONE-D-20-29199_R1_reviewer1 response to authors.pdf [file pone.0246786.s005.pdf]

The authors made reasonable edits to the manuscript from the original submission and responded to many of the questions or comments. However few of the questions needs further clarification and response from the authors.

The batch report values for NfL needs attention form the authors for the high values of NfL.

My response and suggestions (in red font) as below:

1. The authors use the term "cerebral biomarkers" (lines 80 and 86) for S100B, NSE, NfL and tau while there are emerging evidence that elevated levels of NfL are a nonspecific marker of neuronal degeneration and do not tell about the underlying cause. I would be cautions of stating them as cerebral biomarkers and rather use the term biomarkers instead of cerebral biomarkers.

   We thank the reviewer for this comment. We understand that cerebral biomarkers might not be the perfect expression but in the light of there being so many different biomarkers in preeclampsia we would like to keep the expression cerebral biomarkers since it has also been used in previous work and distinguishing biomarkers reflecting the brain in preeclampsia from biomarkers reflecting angiogenesis and placental dysfunction. If the reviewer prefers another name for this group of biomarkers reflecting cerebral involvement, we would be happy to change to that expression.

Thanks for recognizing that cerebral biomarkers might not be the perfect expression. I would still suggest the authors to use an alternative expression or terminology but try to avoid the term 'cerebral biomarkers'.

2. In the current section the authors briefly mention about their previous work (lines 80-82). I would like the authors discuss or provide more background or rationale on why they choose the above four biomarker to study preeclampsia. This would keep the readers more engaged to the current work and create a flow.

   We thank the reviewer for this comment and have now added a section about the background and the section now reads;

   "S100B was the first cerebral biomarker that was reported to be increased in plasma in preeclampsia with severe features by our group and others and in addition, women with visual disturbances demonstrated an increased plasma concentration of S100B. Following these initial reports, there were further studies reporting increased peripheral concentrations also of NSE, NfL and tau, reflecting neurons and axons respectively."

   (page 5, lines 80-85)

   Thanks for making the suggested changes.

Methods Section:

1. The methods section is confusing. In the lines 120-121 the authors mention that the concentrations of tau and NfL in plasma and CSF were measured using the NF-light and Total Tau 2.0 kit on the Simoa platform. But in the lines 128-130 the CSF Total-tau (T-tau) was measured using the INNOTEST ELISA, and CSF NfL was measured using an in-house ELISA. this statement accurate? Why was the CSF T-tau and NfL measured twice using

different platforms? Also, what Simoa platform did the authors use (HD1 or HD-X)? Please clarify.

We apologize for this confusion and this has now been corrected in the methods section and it now reads;

"The concentrations of tau and NfL in plasma were measured using the NF-light and Total Tau 2.0 kits using the Simoa HD-1 platform (Quanterix, Billerica, MA) as previously described in detail"

(page 7, lines 123-125)

Thanks for the edits, the revised section makes sense.

2. For Simoa analysis the calibrators and quality controls (high and low) are run in duplicates. These are in buffer matrix (the calibrators are in some kind of proprietary buffer from Qaunterix) and usually the duplicate measurements have a %CV of <10-20% (which are acceptable range) on Simoa. One of the serious issue I found is that the authors measured the samples in singlicates (as mentioned in lines 123 and 127). The CSF and plasma are true biofluids and I think a duplicate measurement is required at the minimum to assess the experimental integrity and account for the SD (which is not reported). The % CV is cannot established for any of the samples. This specially applies for the ELISA (less sensitive method) tests where duplicate measurements are required.

We thank the reviewer for this important question. The CVs reported were from two quality control (QC) samples (real plasma samples) that were run in duplicates in the beginning and the end of each run. For NfL, between-run precision was 6.0% at 8.5 pg/mL and 5.1% at 121 pg/mL, while for T-tau, between-run precision was 7.3% at 32.2 pg/mL and 7.0% at 7.5 pg/mL. For ELISA:s, between-run precision was below 10% for all assays. This is now added to the methods and now reads;

"Two quality control (QC) samples from plasma were run in duplicates in the beginning and the end of each run."

(page 7, lines 126-127)
and
"Elecsys- and ELISA-based methods were chosen for biomarkers for which Simoa ultrasensitivity was not needed. Between-run precision was <10% for all these assays."
(page 7, lines 133-135)

- Thanks for the clarification. However my question still remains. Why the samples were only measured in singlicates (Page 7, Line 125)?
- Can the authors confirm if the ELISAs were run in singlicates too? If so, this is not common practice for ELISA experiment.
- The two quality control samples used were from plasma (matrix)? Are the authors saying that the controls supplied by Quanterix in the kit are plasma controls? The controls supplied are in buffer matrix. I would suggest the authors to correct the statement w.r.t the controls (these are not plasma controls, refer to Quanterix).
- There are no true controls for plasma used in the Simoa assay in the current study. The

basal or endogenous levels of these biomarkes are not established. This is why a control (non-pregnant women and age matched) would be highly recommended.

3. Why did not the authors include a true control for CSF and plasma? Example: Normal (non-pregnant, age and gender matched CSF and plasma). The reason why I ask is this can serve as a background or endogenous levels for the tested biomarkers in the respective matrix (CSF and plasma). Can the authors comment or show data (either performed by them or any other literature) for the baseline levels of these markers in a non-pregnant women This experiment is critical to corroborate the current results from this study. This is also the reason why I suggested not to use the term cerebral biomarkers as above (Point 1 of the introduction section).

We thank the reviewer for this comment. In this work we concentrated on pregnant women, in particular since it would have been difficult to obtain CSF from non pregnant women of the same age (women in the study were sampled at the time of caesarian section). Plasma concentrations of S100B and NSE were measured in a longitudinal case control study where non pregnant concentrations remained increased in women with previous preeclampsia compared to previous normotensive pregnancies, although the difference was attenuated compared to during pregnancy (NSE 5.07 vs. 4.28 microg/l, $P < 0.05$ and S100B 0.07 vs. 0.06 microg/l, $P < 0.05$).

Thanks for the response. I understand that CSF source is impossible to get for the age and sex matched subjects. How about plasma samples? I suppose this should be fairly easy source from repositories. My concern is that the endogenous levels for these tested biomarkers in plasma (or blood) will establish a basal levels in non-pregnant women or pregnant women. Measuring the changes or fluctuations in the biomarker levels above or below the basal levels would be interesting to study. Some markers have a basal levels with a range which is very broad. Unless these tested biomarkers are not compared to the basal levels the full scope of the study is very limited. Having said that my question still remains 'can the authors comment or show data (either performed by them or any other literature) for the baseline levels of these markers in a healthy and non-pregnant women'.

4. The authors state that all measurements were performed in one round of experiments using a single batch of reagents. While this is a good practice to do, this is also a limited practice as it does not tell about the inter-day variations of the biomarkers tested. Did the authors care to perform an inter-day test to determine the assay variations? This specifically happens with Simoa where the controls (low and high) don't exactly read the same and Quanterix gives a range (not a specific number). Since the sample size is low and on top the authors ran samples in singlicates, a day to day variation would cause certain degree of changes in the measurements.

We thank the reviewer for the comment about this. Whilst this was not relevant to our study, the inter-day variation for our plasma NfL and T-tau measurements are typically below 10%.

This question still remains specially when the samples are measured in singlicates (Page 7, Line 125) and the controls (in buffer) have a range of values not a specific value (see kit insert from Quanterix, the controls have range of values as determined by the manufacturer). The controls are assigned range due to day to day variations. If the samples were run in replicates of two this question would not have raised. (Please refer above).

5. I would be interested to look at the Simoa raw data which can be generated from the batch calibration report. Can I request the authors to share this report?

We have attached the requested batch reports from the runs. The batch reports also include some sample matrix comparisons of no relevance to the current study but these were impossible to delete.

- Thanks for uploading this report.
- The main issue I found with this report is that the NfL concentration values (pg/mL) of plasma (in fact values for all sample barcode, column 1) are sky high. These several times higher than the highest Calibrator (Calibrator H). Which tells that the measurement of samples needs further dilution and the current values are just the extrapolated values and not the precise values. Also, I am not quite sure if these values in batch report are so high, how can the Figure-1 A have low values? Please clarify. This is a critical concern that the authors need to address.

Results Section:

1. While the results are interesting, can the authors comment on why they see (in CSF) increase for some biomarkers (NfL) and decrease for some biomarkers (NSE and tau) compared to the compared to women with normal pregnancies? Though some explanation is offered in the lines 324-327, I did not find this compelling. What does a decrease in biomarker happen in a clinical sense?

We thank the reviewer for pointing this out. We were also surprised by this findings and searching the literature, we don't find any convincing reason to why this is. The only study conducted previously also showed decreased CSF concentrations of tau. As we wrote, one reason could be reduced neuronal activity. In preeclampsia, there are some findings of impaired cognitive function and on long-term, an increased risk of dementia and stroke.

Except for tau, this is the first time these biomarkers are measured in CSF in preeclampsia. We are planning to to similar measurements in CSF and plasma in a population of women with more severe disease (eclamptic fits) to investigate if this pattern holds and what it might depend on. We have added a phrase about this and it now reads as follows;

"There are reports about impaired cognitive function and increased risk of dementia after preeclampsia but if this related to decreased CSF concentrations of tau and NSE remains to be proven." ……. "These findings need to be confirmed in future studies."
(page 17, lines 334-336 and 340-341)

Thanks for adding the above phrase. I understand that the results are intriguing and the authors observe decrease of above mentioned biomarkers which is hard to explain, but can the authors comment on what does a decrease in biomarker happen in a clinical or medical sense?

2. Also can authors comment on why the S100B change (increase) was noticed in plasma but not serum? And similarly (no difference) for NSE and tau?

We assume that the reviewer means CSF and not serum in relation to plasma. S100B is not only produced in astrocytes but also in extra-cerebral tissues such as muscle cells and adipose tissue. Therefore, the increased peripheral concentrations could be caused by an increased secretion from fat or muscle cells. This has now been stressed and reads as follows;

"Our results showed that S100B was increased in plasma but not in CSF in preeclampsia. This could have different explanations. One reason could be that the signal from S100B produced in other extracerebral tissues such as muscle cells and fat cells are dominating causes to increased plasma concentrations in preeclampsia. Another explanation could be that S100B, produced in astrocytic end-feet close to the blood brain barrier (BBB), is secreted in higher amounts into the blood stream due to BBB injury and thus depleted from the CNS."
(page 16, lines 310-315)

My apologies, I meant CSF. Thanks for adding the above statement. The reasons sound plausible. It would be a good idea to add in the narrative and provide a citation for the basal levels of S100B in normal pregnant women? This goes inline with the above for endogenous levels.

3. Table 2. Why is the units for S100B and NSE denoted in ug/L, while for NfL and tau the units are in pg/mL? Is there a reason why the authors choose to do so? It would be better to see all units in pg/mL, for uniformity in the table.

We thank the reviewer for this comment. 1 pg/ml = 0.001 ug/L and due to the differences in concentrations we chose to keep these units to make it easier to read. If the reviewer still feels the same units would be better, we could change but then the concentrations of S100B and NSE would be very high.

Thanks for the clarification. In that case no change is needed.

4. I am not sure how the hemolysis was observed (line 222) only for seven women in each group. Aren't the same (aliquot/stock) CSF was used all the other biomarker measurement? In that case all the CSF measurements for other biomarkers (for seven women) should have the hemolysis. Please clarify or am I missing something here?. Also for the lines (304-305)

We apologize for the confusion. The hemolyzed samples were only removed for the NSE analyses since NSE is produced in red blood cells and if NSE would be analyzed in hemolyzed samples, the signal from red blood cells would over-ride any other signal.

5. I understand that the authors could not perform any stats on the woman with preeclampsia and cerebral edema. But can the authors comment on why the CSF biomarkers (NSE and tau) which was decreased for the preeclampsia group (compared to normal group), is increased for this particular subject? And the same applies for the S100B.

We thank the reviewer for this important comment. As discussed above, it is not yet known why tau and NSE are decreased in CSF in preeclampsia on a group level, though these groups are small and findings need to be repeated in larger sample sizes with different phenotypes of preeclampsia. Our speculation in this case would be that a woman with cerebral edema has a larger cerebral insult and thus a potential increased neuronal, glial and axonal injury. Cerebral

edema in preeclampsia is thought to be both vasogenic and sometimes cytotoxic.
The cytotoxic edema could potentially be non-reversible, causing persistent injury and this could be the cause to the increased concentrations. Though since this was just one case we chose not to put to much emphasis on that in the discussion section but we look forward to coming analyses of biomarkers in CSF in women with clinical and radiological cerebral complications such as eclampsia and PRES that we are planning for in the coming 6 months.

Thanks for making the edits. I would suggest the authors to add part this statement in the limitations section.

Discussion Section:

1. I truly found the lines 286-288 overstated. The results show CSF concentrations of NfL were increased in preeclampsia but there was no evidence presented to indicate any neuro- axonal injury. Are the authors inferring increased NfL in preeclampsia as direct outcome of neuro-axonal injury while the MRI show normal readings? I would re-write this section.

   We thank the reviewer for pointing this out. As for MRI imaging, only larger deficits can be detected such as cerebral edema in the acute phase. There are other MRI modalities such as ASL and IVIM to investigate cerebral perfusion and white matter integrity but those were not used in this particular study. NfL is a sensitive marker for axonal degeneration and the fact that this biomarker is increased both in CSF and plasma in preeclampsia, in combination with the known longterm cerebral effects after preeclampsia such as dementia and cognitive deficits strengthens this finding. We have, according to the reviewer's wish, modified this section a bit and it now reads;

   "These findings might indicate a neuro-axonal injury in preeclampsia even when clinical and radiological neurological complications are absent."

   (page 15, lines 293-294)

   Thanks for making the edits.

2. I think the authors should comment and/ or mention in the limitations section on why they could not reproduce earlier findings of increased NSE and tau plasma concentrations in preeclampsia as their older studies (lines 294-297). Similarly the statements made in lines 309-312 can be moved to study limitations rather.

   We thank the reviewer for this comment and have moved these sections to "Limitations" and in addition added a section about the fact that earlier findings could not be reproduced in our population and reads;
   "Finally, our findings could not support earlier reports of increased plasma concentrations of tau and NSE in preeclampsia. This could be due to a smaller number of women in our study or other phenotypes of preeclampsia compared to other studies since preeclampsia is a heterogenous disorder."
   (page 18, lines 365-368)

   Thanks for making the edits.

3. A citation is required of the line 298.

Citations are now added.

Thanks for adding the citation.

4. Can the authors support the claim that NfL extensive marker for multiple sclerosis and traumatic brain injury? Is the claim of increased NfL in preeclampsia in the current study is inferred form the above study? Please explain (this goes with the point 1 above).

   We have now added references for this statement in the discussion section (ref 29-30)

   Thanks for making the edits.
5. Lines 308-312 there are too much speculation from the authors. I suggest to re-construct this section to tie it to the current study theme.

   The section has now been reconstructed and now reads;

   "The reason for this is not known. We speculate about that the NSE signal in plasma in this study might be derived from other sources such as red blood cells which might also have impacted previous reports of peripheral concentrations of NSE in preeclampsia."
   (page 16, lines 318-321)

   Thanks for making the edits.

6. The strength and limitation sections I think is not well written and requires reconstruction. The above limitation (see above) should be incorporated.

   The above limitations have now been incorporated into this section as suggested.

   Thanks for making the edits.

7. Does the MRI examinations reflect a degree of cerebral injury (as stated in lines 347-348)? I don't see it in Table 3. Please clarify.

   We apologize for the confusion. What we meant was that by using MRI, we could characterize the population better in terms of evidence of cerebral edema and white matter lesions. In our population, there were no difference between groups. We have now changed this to;
   "the availability of MRI examinations to characterize cerebral involvement"
   (page 18, lines 359-360)

Conclusion section:

1. Lines 352-353 is overstated without any direct/tangible evidence for neuroaxonal injury I would refrain from using such sentence without a clear confirmation.

   We have now rephrased the section and it now reads;
   "We have shown that NfL has potential to be the most promising cerebral biomarker to reflect possible neuroaxonal injury in preeclampsia"

   (page 19, lines 371-372)
   - Can I debate that extracerebral tissues (such as muscle cells and fat cells, as stated as a

reason by authors as above for increased S100B) may also be acting as a dominating causes to increased plasma concentrations in preeclampsia? Especially since the authors are using ultrasensitive Simoa assays for NfL measurement and also now we can see that the batch report has high NfL (pg/mL) values. Can I get a comment form the authors if my argument is reasonable?

- I would also suggest the authors to omit the words most and cerebral and instead use 'NfL has potential to be the promising biomarker'…..

2. Lines 353-354 is confusing to me. What does the author mean here? Please clarify.

We apologize for the confusion. We realize this phrase is unecessary and it has been removed.

Thanks for making the edits.

3. Lines 357-358 again is overstated. It is not clear how increased CSF concentrations point to a neuroaxonal injury in preeclampsia and how does detection and facilitate treatment? Does the authors mean increased NfL is a diagnostic marker for preeclampsia?
I sincerely request the authors to reconstruct the whole section without these overstatement. I would appreciate any direct evidence for neuroaxonal injury as the authors state here.

We have rephrased a lot of the conclusion as per above comments and it now reads;
"We have shown that NfL has potential to be the most promising cerebral biomarker to reflect possible neuroaxonal injury in preeclampsia. Though our findings originate from a small study sample and need to be further investigated both in relation to preeclampsia with clinically evident neurological injury such as eclampsia and cerebral edema as well as more subtle neurological impairment in preeclampsia such as neurocognitive deficiencies. Should further studies demonstrate increased NfL concentrations in plasma in cases of preeclampsia with neurological impairment, NfL could potentially have a role as prognostic and /or diagnostic biomarker of cerebral involvement in preeclampsia."
(page 19, lines 371-378)

Thanks for making the edits. Please refer to above comment or argument and also w.r.t omitting words most and cerebral…

Figure-1
The axis titles of the figure are too small. I would suggest the authors to increase the font size for better resolution.

The axis titles are now increased in font size and also numbers and overall titles.

Thanks for making the edits
