# Supplementary material for: Signs of neuroaxonal injury in preeclampsia—A case control study
Source: PLoS One. 2021 Feb 8;16(2):e0246786. doi: 10.1371/journal.pone.0246786 (PMC7869986; doi:10.1371/journal.pone.0246786)
Supplement: Attachment — Submitted filename: Response to reviewers.docx [file pone.0246786.s006.docx]

*Dear Dr Kobeissy,*

*Re: PONE-D-20-29199R1; Signs of neuroaxonal injury in preeclampsia – a case control study*

*We thank the reviewers for their comments. Here we are pleased to provide our responses. We look forward to further correspondence from PLoS ONE. All changes to the manuscript are marked in yellow.*

*In addition to the reviewer’s comments, we have also noted an error in the text. Concerning S100B, this has been analysed in serum, not plasma. This has now been corrected throughout the text and highlighted in yellow.*

Kind regards,

Dr Lina Bergman, corresponding author

**Reviewer 1**

The authors made reasonable edits to the manuscript from the original submission and responded to many of the questions or comments. However few of the questions needs further clarification and response from the authors.

The batch report values for NfL needs attention from the authors for the high values of NfL.

My response and suggestions as below:

**Introduction**

1) Thanks for recognizing that cerebral biomarkers might not be the perfect expression. I would still suggest the authors to use an alternative expression or terminology but try to avoid the term ‘cerebral biomarkers’.

*We thank the reviewer for this comment. As previously mentioned, this term has been used for these biomarkers in other published papers in the area and since it is a term that we need to repeat throughout the manuscript and need a common expression for, we find it hard to exclude it. If the reviewer has other suggestions, we would be happy to look into that. But since preeclampsia is a disease affecting many different organ systems where multiple biomarkers are searched for, such as angiogenic biomarkers, endothelial biomarkers, renal biomarkers etc, we strongly feel we have to provide an expression for biomarkers specifically investigated to reflect cerebral involvement in preeclampsia.*

**Methods Section**

2) Thanks for the clarification. However my question still remains. Why the samples were only measured in singlicates (Page 7, Line 125)?

*We thank the reviewer for this additional comment. As specified in the manuscript, the analytical variation of the assays is low enough (<10%) to allow for singlicate measurements. Duplicate measurements would not add much and would also result in higher consumption of sample volumes.*

3) Can the authors confirm if the ELISAs were run in singlicates too? If so, this is not common practice for ELISA experiment.

*Whether samples are run as singlicates or duplicates does not depend on the analytical method as such but the precision of the method. The low analytical variation for all the immunoassays allowed us to do singlicate measurements for all analytes.*

4) The two quality control samples used were from plasma (matrix)? Are the authors saying that the controls supplied by Quanterix in the kit are plasma controls? The controls supplied are in buffer matrix. I would suggest the authors to correct the statement w.r.t the controls (these are not plasma controls, refer to Quanterix).

*We are sorry for any confusion. We are saying that we always create our own QC samples to monitor analytical variation across plates and runs in addition to the controls supplied by the kit manufacturer. The precision data presented in the manuscript are data from our own QC samples that were composed of plasma, as stated in the manuscript.*

5) There are no true controls for plasma used in the Simoa assay in the current study. The basal or endogenous levels of these biomarkes are not established. This is why a control (non-pregnant women and age matched) would be highly recommended.

*We thank the reviewer for this comment. As far as we know, no studies have yet published on NfL and tau in the same population with pregnant and non pregnant women. This is beyond the scope of the current study and not important for the research questions addressed here, but we have another manuscript under preparation that deals with this question and that will be published in 2021.*

6) Thanks for the response. I understand that CSF source is impossible to get for the age and sex matched subjects. How about plasma samples? I suppose this should be fairly easy source from repositories. My concern is that the endogenous levels for these tested biomarkers in plasma (or blood) will establish a basal levels in non-pregnant women or pregnant women. Measuring the changes or fluctuations in the biomarker levels above or below the basal levels would be interesting to study. Some markers have a basal levels with a range which is very broad. Unless these tested biomarkers are not compared to the basal levels the full scope of the study is very limited. Having said that my question still remains ‘can the authors comment or show data (either performed by them or any other literature) for the baseline levels of these markers in a healthy and non-pregnant women’.

*We thank the reviewer for this additional comment. For S100B and NSE, there are non pregnant data from one year after pregnancy as previously reported. Plasma concentrations of S100B and NSE were measured in a longitudinal case control study where non pregnant concentrations remained increased in women with previous preeclampsia compared to previous normotensive pregnancies, although the difference was attenuated compared to during pregnancy (NSE 5.07 vs. 4.28 microg/l, P < 0.05 and S100B 0.07 vs. 0.06 microg/l, P < 0.05). This paper is cited as number 10 in the reference list for further information.*

*In addition, as mentioned above, we are currently analyzing data for all four biomarkers in a cross sectional study comparing women with preeclampsia, normotensive women and non pregnant women but this is yet to be published. Other than that, we have no other repositories of non-pregnant values in the same population / batch of analyses.*

7) This question still remains (inter-day variation) specially when the samples are measured in singlicates (Page 7, Line 125) and the controls (in buffer) have a range of values not a specific value (see kit insert from Quanterix, the controls have range of values as determined by the manufacturer). The controls are assigned range due to day to day variations. If the samples were run in replicates of two this question would not have raised. (Please refer above).

*The precision data presented in methods cover the total variation of each biomarker at two clinically relevant biomarker concentrations in the relevant sample matrix (plasma) when the samples were analysed (please see Methods).*

8) Thanks for uploading this report (SIMOA raw data). The main issue I found with this report is that the NfL concentration values (pg/mL) of plasma (in fact values for all sample barcode, column 1) are sky high. These several times higher than the highest Calibrator (Calibrator H). Which tells that the measurement of samples needs further dilution and the current values are just the extrapolated values and not the precise values. Also, I am not quite sure if these values in batch report are so high, how can the Figure-1 A have low values? Please clarify. This is a critical concern that the authors need to address.

*We fail to see the reviewer’s concern. The critical parameter to consider is the AEB value; within the range of the calibrator AEBs and none is higher than Calibrator H. Could the reviewer have misinterpreted the report? Please let us know if we could clarify anything further.*

**Results section**

9) Thanks for adding the above phrase (*There are reports about impaired cognitive function and increased risk of dementia after preeclampsia but if this related to decreased CSF concentrations of tau and NSE remains to be proven.” ……. “These findings need to be confirmed in future studies.)*. I understand that the results are intriguing and the authors observe decrease of above mentioned biomarkers which is hard to explain, but can the authors comment on what does a decrease in biomarker happen in a clinical or medical sense?

*In preeclampsia, it is not clear why tau and and NSE would be decreased in CSF. We refer to the present text in the manuscript that reads;*

“An unexpected finding was that women with preeclampsia had reduced CSF concentrations of NSE and tau. In the absence of neurodegeneration, extracellular levels of tau are regulated by neuronal activity-dependent release of the protein, which is likely to determine the corresponding CSF concentrations. It is possible that such a mechanism regulates extracellular NSE concentrations as well. Reduced CSF concentrations of tau and NSE in preeclampsia could thus reflect reduced neuronal activity.”

(page 17, lines 335-340)

*We are sorry that we are not able to provide further reflections on why we find these biomarkers in reduced concentrations in the CSF but we hope that future data can elucidate this when investigating more severe forms of the disease and also in relation to neuroimaging findings of cerebral edema.*

10) Also can authors comment on why the S100B change (increase) was noticed in plasma but not serum? And similarly (no difference) for NSE and tau?

*We assume the reviewer refers to CSF. We have elaborated on this according to below citations;*

*”*Our results showed that S100B was increased in plasma but not in CSF in preeclampsia. This could have different explanations. One reason could be that the signal from S100B produced in other extracerebral tissues such as muscle cells and fat cells are dominating causes to increased plasma concentrations in preeclampsia. Another explanation could be that S100B, produced in astrocytic end-feet close to the blood brain barrier (BBB), is secreted in higher amounts into the blood stream due to BBB injury and thus depleted from the CNS.”

(page 16-17 lines 314-319)

”We speculate about that the NSE signal in plasma in this study might be derived from other sources such as red blood cells which might also have impacted previous reports of peripheral concentrations of NSE in preeclampsia.

Regarding plasma concentrations of tau, there were no significant differences between groups. In addition, the correlation between plasma concentrations and CSF concentrations of tau in Alzheimer’s disease has proven to be weak and other confounding sources of tau have to be considered. These findings are supported in this study where we did not find any correlation between CSF and plasma concentrations of neither tau, NSE or S100B. Thus, alternative extracerebral sources of tau, NSE and S100B in preeclampsia have to be considered.”

(page 17, lines 325-334)

11) My apologies, I meant CSF. Thanks for adding the above statement. The reasons sound plausible. It would be a good idea to add in the narrative and provide a citation for the basal levels of S100B in normal pregnant women? This goes inline with the above for endogenous levels.

*We have now added this to the manuscript and it now reads;*

“Circulating concentrations of S100B in non pregnant women are reported at around 0.06 ug/L.”

(page 17, lines 320-321)

12) Thanks for making the edits. I would suggest the authors to add the statement about the woman with cerebral edema in the limitations section.

*We thank the reviewer for this suggestion and have now modified and added this section which reads as follows;*

”It is not yet known why tau and NSE were decreased in CSF in preeclampsia on a group level, though these groups are small and findings need to be repeated in larger sample sizes with different phenotypes of preeclampsia. The only woman with cerebral edema demonstrated increased CSF concentrations of all biomarkers. Our speculation would be that a woman with cerebral edema has a larger cerebral insult and thus a potential increased neuronal, glial and axonal injury. Cerebral edema in preeclampsia is thought to be both vasogenic and sometimes cytotoxic.The cytotoxic edema could potentially be non-reversible, causing persistent injury and this could be the cause to the increased concentrations.”

(page 19, lines 369-376)

**Conclusion section:**

13) Can I debate that extracerebral tissues (such as muscle cells and fat cells, as stated as a reason by authors as above for increased S100B) may also be acting as a dominating causes to increased plasma concentrations in preeclampsia? Especially since the authors are using ultrasensitive Simoa assays for NfL measurement and also now we can see that the batch report has high NfL (pg/mL) values. Can I get a comment form the authors if my argument is reasonable?

*We thank the reviewer for this comment. Previous literature has mainly reported on median concentrations between group and not relating this to intracerebral engagement. Some studies have reported on correlations to neurological symptoms. Thus, this has been the first study to make an effort to relate the peripheral concentrations of these biomarkers to corresponding concentrations in the CNS through CSF concentrations and also to MRI findings. Since this cohort of women did not experience any clinically evident neurological complications, the interpretation must still be cautios but points towards NfL as the most promising biomarker as mentioned in the main text. We agree with the reviewer that peripheral concentrations of S100B, NSE and tau might be depending on contributions from alternative sources and this was the main aim of the paper to add to previous literature.*

14) I would also suggest the authors to omit the words most and cerebral and instead use ‘NfL has potential to be the promising biomarker’…..

*We are sorry but we don’t agree with the reviewer in this since there are other promising biomarkers for preeclampsia to predict onset of disease and also severity of disease. Though, there are not yet any clinically available objective biomarkers of cerebral involvement in preeclampsia and since preeclampsia is a heterogenous disorders where only a small proportion of women diagnosed will be affected by cerebral complications, there is a need for specific biomarkers for cerebral engagement. If the reviewer wish to replace ”cerebral” with another word reflecting CNS involvement we would be happy to consider to change but we can not only replace it with ”biomarker”.*

15) Please refer to above comment or argument and also w.r.t omitting words most and cerebral…

See response above.
