# Supplementary material for: Signs of neuroaxonal injury in preeclampsia—A case control study
Source: PLoS One. 2021 Feb 8;16(2):e0246786. doi: 10.1371/journal.pone.0246786 (PMC7869986; doi:10.1371/journal.pone.0246786)
Supplement: Attachment — Submitted filename: Response to reviewers.docx [file pone.0246786.s007.docx]

*Dear Dr Kobeissy,*

*Re: PONE-D-20-29199R2; Evidence of neuroaxonal injury in preeclampsia – a case control study*

*We thank the reviewers for their comments. Here we are pleased to provide our responses. We look forward to further correspondence from PLoS ONE. All changes to the manuscript are marked in yellow.*

Kind regards,

Dr Lina Bergman, corresponding author

**Editor Comments**

Dear Dr. Bergman,
I apologize that the review process took that long, the second reviewer have few minor comments that I think can be easily addressed. Once answered, the paper is not going through another round of review. I will be accepting it. Thank you so much and congrats on this elegant work

*We thank the editor for these kind words and are delighted to hear that you appreciate our work.*

**Reviewer 1**

1) Thanks for the revised manuscript. However few of the response from the authors is not satisfactory and I am inclining to suggest revisions again. With regards to the batch report, I offer my apologies for the interpretation w.r.t the calibrators. Perhaps the data format in which it was presented (with the significant digits and a comma instead of period) created the confusion. This confusion is now resolved.

*We are happy that the issue is now resolved.*

2) This is a preliminary data and the authors do acknowledge that no studies have yet published on NfL and tau in the same population with pregnant and non-pregnant women. Most of the data is reasonable but the interpretation with NfL is slightly exaggerated. This initial data should be looked at very cautiously as the authors claim that NfL as “most promising cerebral biomarker”. This overemphasis without direct evidence of cerebral damage/involvement and that extra cerebral tissues (muscle cells and fat cells, as stated as a reason by authors for increased S100B) may also be acting as a dominating causes to increased plasma concentrations in preeclampsia. My argument is that other factors could also have affected biomarkers (considering the pregnant conditions) and use of an ultra-high sensitive assay (Simoa). Besides no controls (non-pregnant women) were used and therefore no Simoa NfL data is available from the authors for the range of basal levels of NfL in these age matched non-pregnant women.

*We thank the reviewer for this comment. Indeed, there might be several different sources of errors for most of the biomarkers used in medicine where none is 100% specific or sensitive for a condition. Though we still argue that there is quite a few published articles on all four biomarkers presented in this article in pregnancy and preeclampsia so this is not the first time to demonstrate increased circulating concentrations of NfL, tau, NSE and S100B in preeclampsia. Though it is the first time, to our knowledge, that data regarding CSF concentrations of these biomarkers is presented in preeclampsia. This was the main purpose of this paper in order to strengthen the cerebral origin and compare peripheral concentrations to CSF concentrations to rule in/rule out if the increased plasma concentrations truly reflected also increased CSF concentrations. This only held true for NfL and in addition, NfL is quite brain specific (*[*https://www.proteinatlas.org/ENSG00000277586-NEFL/tissue*](https://www.proteinatlas.org/ENSG00000277586-NEFL/tissue)*). Therefore, we would still like to argue that our findings of increased peripheral NfL concentrations most probably reflects increased secretion of NfL from the brain in preeclampsia.*

*We are currently preparing an article about peripheral concentrations of NfL in non pregnant women as a reference group but that is another population and will hopefully be published during 2021. In this study, plasma concentrations of NfL are increased in preeclampsia compared to both normotensive pregnancies and non pregnant women. Even so, we argue that the results from this study can stand alone and that a reference group of normotensive pregnancies is enough in this case. In addition, as argued before, it is almost impossible to obtain CSF samples from healthy non pregnant women of the same age since these samples were obtained at time of spinal anesthesia for a caesarian section. We have put a lot of emphasize in the discussion regarding alternative sources that will always be a confounder to the results.*

3) Subsequently, several recent studies are emerging to indicate NfL as a concussion biomarker, one should be excessively cautious of the statements used. I think this work is important and should be published, but I think the overemphasis on terms like ‘cerebral’ and ‘most’ promising biomarker without a direct evidence in the manuscript may lead to the results being misinterpreted and misrepresented. As such I emphasize to avoid them. I cannot provide with an alternate expression other than not using the words cerebral and most.

*We have understood that the reviewer would like us to replace the words ”cerebral” and ”most”. Regarding ”cerebral” as explained in earlier responses to reviewers, we can’t find a more suitable expression and in addition, the phrase is commonly used in similar papers describing these biomarkers in preeclampsia. We welcomed an alternative expression but understand that the reviewer also can’t find a more appropriate way of expressing this group of biomarkers. Therefore, we can’t see how we can change this to another phrasing.*

*Regarding the expression ”most” we have now, to the reviewer’s request, replaced ”the most” with ”a” in the conclusion in the abstract and in the main text. We think the message in the manuscript is quite clear regarding the need for larger studies of women with clinically evident cerebral complications to confirm these findings.*

Page 4, line 60 and page 19, line 386

4) Also, I would like to differ with the authors reasoning for not running duplicate samples. It might be appealing (for whatever reasons) to run each sample in only a single, but running samples in duplicate allows for the calculation of sample variation and provides a measure of the precision of the assay (which cannot be made for the current experiment without inter-day experimental data for the samples). And also the technical error can usually be identified as an outlier well, and that well can be removed from analysis. It is very important to run standards and blanks in duplicate or triplicate to be certain of the precision of these critical measurements. So, I would also suggest the authors to state this under study limitations. I still feel that this data is valuable and worth reporting, but I think response to above two questions is warranted.

*We thank the reviewer for this comment. Though we don’t agree since these analyses are well established and reliable, please see previous comments to the reviewer. Therefore, we think it would be unnecessary to run all of them in duplicates, in particular regarding the small volumes of CSF and the expected outomes of running duplicates (rendering similar results). In contrary, one could argue that if one has a reliable instrument it would not be a reasonable priority to use funds and sample volume to run duplicates if these research funds and biological samples could be used to answer further research questions, using the samples and funds in the most efficient way.*

*The analytical variation of the assay is monitored using quality control samples as clearly specified in Methods.*
